# Prenylation inhibitors stimulate both estrogen receptor α transcriptional activity through AF-1 and AF-2 and estrogen receptor β transcriptional activity

**DOI:** 10.1186/bcr956

**Published:** 2004-11-08

**Authors:** Philippe Cestac, Guillaume Sarrabayrouse, Claire Médale-Giamarchi, Philippe Rochaix, Patrick Balaguer, Gilles Favre, Jean-Charles Faye, Sophie Doisneau-Sixou

**Affiliations:** 1 Département 'Innovation Thérapeutique et Oncologie Moléculaire', Centre de Physiopathologie de Toulouse Purpan, INSERM U563 and Institut Claudius Regaud, Toulouse, France; 2 INSERM 540, Endocrinologie Moléculaire et Cellulaire des Cancers, Montpellier, France

**Keywords:** estrogen receptor, farnesyltransferase inhibitor, geranylgeranyltransferase inhibitor, Rho proteins, transcription

## Abstract

**Introduction:**

We showed in a previous study that prenylated proteins play a role in estradiol stimulation of proliferation. However, these proteins antagonize the ability of estrogen receptor (ER) α to stimulate estrogen response element (ERE)-dependent transcriptional activity, potentially through the formation of a co-regulator complex. The present study investigates, in further detail, how prenylated proteins modulate the transcriptional activities mediated by ERα and by ERβ.

**Methods:**

The ERE-β-globin-Luc-SV-Neo plasmid was either stably transfected into MCF-7 cells or HeLa cells (MELN cells and HELN cells, respectively) or transiently transfected into MCF-7 cells using polyethylenimine. Cells deprived of estradiol were analyzed for ERE-dependent luciferase activity 16 hours after estradiol stimulation and treatment with FTI-277 (a farnesyltransferase inhibitor) or with GGTI-298 (a geranylgeranyltransferase I inhibitor). In HELN cells, the effect of prenyltransferase inhibitors on luciferase activity was compared after transient transfection of plasmids coding either the full-length ERα, the full-length ERβ, the AF-1-deleted ERα or the AF-2-deleted ERα. The presence of ERα was then detected by immunocytochemistry in either the nuclei or the cytoplasms of MCF-7 cells. Finally, *Clostridium botulinum *C3 exoenzyme treatment was used to determine the involvement of Rho proteins in ERE-dependent luciferase activity.

**Results:**

FTI-277 and GGTI-298 only stimulate ERE-dependent luciferase activity in stably transfected MCF-7 cells. They stimulate both ERα-mediated and ERβ-mediated ERE-dependent luciferase activity in HELN cells, in the presence of and in the absence of estradiol. The roles of both AF-1 and AF-2 are significant in this effect. Nuclear ERα is decreased in the presence of prenyltransferase inhibitors in MCF-7 cells, again in the presence of and in the absence of estradiol. By contrast, cytoplasmic ERα is mainly decreased after treatment with FTI-277, in the presence of and in the absence of estradiol. The involvement of Rho proteins in ERE-dependent luciferase activity in MELN cells is clearly established.

**Conclusions:**

Together, these results demonstrate that prenylated proteins (at least RhoA, RhoB and/or RhoC) antagonize the ability of ERα and ERβ to stimulate ERE-dependent transcriptional activity, potentially acting through both AF-1 and AF-2 transcriptional activities.

## Introduction

Both estrogen receptor (ER) subtypes, ERα and ERβ, are ligand-activated transcription factors. ERα is the major ER in mammary epithelium and is an important regulator of cell growth, differentiation and malignant transformation. After binding to estrogen, the receptors associate with specific estrogen response elements (EREs) within the promoters of estrogen-regulated genes or the receptors affect the activity of other transcription factor complexes such as AP-1 (Jun–Fos). The two ER subtypes share affinity for the same ligands and DNA response elements [1]. These nuclear receptors consist of six domains including the A/B domain containing the AF-1 autonomous transcription activation domain, the C domain containing the DNA binding domain, the E domain containing the ligand binding domain, and the AF-2 ligand transcription activation domain located in the C terminus of the receptor. Transcriptional activation by ERα is mediated by the synergistic action of the two distinct activation functions; although AF-1 is constitutively active, it is usually weaker than the AF-2 activity. In contrast, ERβ appears to have no significant AF-1 activity and thus depends entirely on the ligand-dependent AF-2 activity [2].

The current model for ER action suggests that the ER modulates the rate of transcription through interactions with the basal transcription machinery and by altering the recruitment of co-activators that modify chromatin organization at the promoter level of target genes [3-5]. In addition, tissue-specific nuclear receptor co-activators and co-repressors have been described that can modify the transcriptional activity of the ER [6-8].

There is increasing evidence, however, that not all the biological effects of estrogens are mediated by direct control of target gene expression; indeed, some effects are attributed to estrogenic regulation of signaling cascades [9-11]. Several rapid effects suggest that estrogens can interact with receptors that are located in close proximity to the plasma membrane [12,13]. These receptors, which appear to form a subpopulation of the classical ER, are associated with the cell membrane and are responsible for several manifestations of estrogenic signaling [14,15]. Recent data explain how the coordinate interactions between a newly identified scaffold protein, MNAR, the ER and Src lead to Src activation, demonstrating the integration of ER action in Src-mediated signaling [11,13]. These data highlight new evidence for a cross-talk between estradiol (E2) and growth-factor-induced cytoplasmic signaling. Several components of these signaling pathways are low molecular weight GTPases, such as Ras, that require prenylation to function.

Ras belongs to the Ras superfamily of low molecular weight proteins. The activity of such proteins is controlled by a GDP/GTP cycle. Members of the Ras superfamily include the Ras, Rho and Rab subfamilies. The Ras and Rho proteins of this superfamily are modified post-translationally by the isoprenoid lipids farnesylpyrophosphate and geranylgeranylpyrophosphate. Farnesyltransferase and geranylgeranyltransferase I respectively catalyze the covalent attachment of the farnesyl group (C_15_) and the geranylgeranyl group (C_20_) to the carboxyl-terminal cysteine of prenylated proteins. Prenylation appears to be essential not only for membrane association, but also for biological activity [16].

In a previous report, we demonstrated the implication of both farnesylated and geranylgeranylated proteins in E2 actions, as prenylation inhibitors block the cell-cycle progression driven by E2 and stimulate the transcriptional activity of the ER [17]. Our data strongly suggest that the transcriptional stimulation of the ER by prenylation inhibitors is due to a shift in the association of a transcription co-regulator with the ERα. Among the numerous co-activators identified to date, one of the best characterized is the p160 family of proteins including SRC-1, GRIP1 and RAC3, which enhance ER transactivation by recruiting other transcriptional regulatory factors such as CREB-binding protein and p300 (reviewed in [18]). We demonstrated that both FTI-277 (a farnesyltransferase inhibitor) and GGTI-298 (a geranylgeranyltransferase I inhibitor) increase the association of the SRC-1 co-activator with ERα and that FTI-277 decreases the association of HDAC1 with ERα, which is essential for transcriptional repression.

In the present report, we assess in further detail the role of prenylated proteins in the estrogenic regulation of transcription in MCF-7 cells and in HeLa cells transiently expressing ERα, ERβ or ERα mutants. Our data clearly outline that prenyltransferase inhibitors (FTI-277 and GGTI-298) only stimulate ERE-dependent luciferase activity in stably transfected MCF-7 cells. They stimulate both ERα-mediated and ERβ-mediated ERE-dependent luciferase activity, without any obvious relocation of the ER from the cytoplasm to the nuclei. By contrast, the roles of both AF-1 and AF-2 of ERα appear to be determining in this effect. These results further establish the involvement of prenylated proteins, and more specifically the Rho-mediated signaling pathway, in the negative regulation of ER transcriptional activity.

## Materials and methods

### Materials

Materials used for cell culture were from InVitrogen Life Technology (Cergy Pontoise, France). Polyethylenimine was purchased from Sigma-Aldrich S.a.r.l. (St Quentin Fallavier, France). FTI-277 and GGTI-298 were a generous gift from S Sebti (University of South Florida, Tampa, FL, USA). Both FTI-277 and GGTI-298 peptidomimetics were dissolved in a solution of 10 mM dithiothreitol in dimethylsulfoxide.

### Cell culture

The MCF-7 human breast adenocarcinoma cell line was obtained from the American Tissue Culture Collection (Rockville, MD, USA). The development of stable transfectants of MCF-7 cells (MELN cells) or HeLa cells (HELN cells) has been described previously [19]. These cells were established by transfecting MCF-7 cells or HeLa cells with the ERE-β-globin-luc-SV-Neo plasmid and therefore expressing luciferase in an estrogen-dependent manner. MCF-7 cells were routinely cultured in RPMI 1640, and MELN cells and HELN cells were cultured in DMEM growth media, supplemented with 5% FCS. Cells were incubated at 37°C in a humidified 5% CO_2 _incubator.

### Cell transfection

Cells were grown for 3 days in phenol red-free medium, containing 5% dextran-coated charcoal-treated fetal calf serum (DCC-FCS). Then 6 × 10^4^cells were seeded per well in 12-well plates and grown for 1 day in phenol red-free medium, containing 5% DCC-FCS. Cells were then transfected (0.4 μg/well DNA, 1 μl/well polyethylenimine in a final volume of 500 μl). Five hours after transfection, and for optimal FTI-277 treatment, cells were pretreated for 24 hours with the farnesyltransferase inhibitor prior to receiving E2 (5 nM) and FTI-277 [17]. For GGTI-298 treatment, no preincubation was performed and the inhibitor was added simultaneously with E2. Control experiments were also carried out; on the one hand in the absence of E2, and on the other hand in the presence of E2 and in the absence of inhibitors.

The plasmids used were the β-glob-Luciferase and the corresponding PGL3 empty vector for MCF-7 transient transfection, pSG5-REβ coding the full-length ERβ, HGO coding the full-length ERα, HG19 coding the AF-1-deleted ERα, HG7 coding the AF-2-deleted ERα, and the pSG5 empty vector for transfection of HELN cells. The HG0, HG19 and HG7 plasmids [20] were provided by Prof Chambon (Institut de Génétique et de Biologie Moléculaire et Cellulaire, Centre National de la Recherche Scientifique, Strasbourg, France). The pRL-CMV Vector (Promega, Charbonnières, France) coding the Renilla Luciferase was co-transfected as a reporter gene.

### Luciferase assay in cellular homogenates

Cells were seeded in 12-well plates as already described, and were treated or not with prenyltransferase inhibitors and E2 for 16 hours in a final volume of 500 μl. At the end of the treatment, cells were washed with PBS and lysed in 100 μl lysis buffer (Promega). The luciferase activity from both Luciferase genes was measured using the Dual Luciferase Reporter (Promega), according to the manufacturer's instructions. For MELN cells that were not transfected with the Renilla Luciferase gene, protein concentrations were measured using the Bradford technique to normalize the luciferase activity data. A minimum of 15,000 relative light units were generated for the control conditions, used as the reference onefold induction. For each condition, average luciferase activity was calculated from the data obtained from three independent wells.

### ER immunocytochemical detection

For each condition, 5 × 10^5 ^MCF-7 cells were seeded onto glass slides in 100 mm Petri dishes and were grown for 6 days in phenol red-free medium, containing 5% DCC-FCS. Cells were then preincubated or not with farnesyltransferase inhibitor and received E2 (5 × 10^-9 ^M) and/or inhibitors 24 hours later. At the end of the treatment period (24 hours), cells were fixed in paraformaldehyde (4% in PBS) for 1 min. The staining was performed by a Techmate Horizon™ slide processor using a one-step peroxidase-conjugated polymer backbone visualization system (En Vision™ DAKO, Glostrup, Denmark) according to the manufacturer's instructions. The chromogenic substrate was 2,3'-diaminobenzidine. The primary antibody used was a monoclonal anti-ERα antibody (clone 1D5; DAKO). Negative controls were performed using the same staining technique without incubation with the primary antibody. Data were quantified using the ImageQuant software (Molecular Dynamics, Inc., Sunnyvale, CA, USA). Results are expressed in arbitrary units for either nuclei or cytoplasms of analyzed cells, in each experimental condition.

### *Clostridium botulinum *C3 exoenzyme treatment

MELN cells were grown for 3 days in phenol red-free medium, containing 5% DCC-FCS. For each condition, 10^5 ^cells were seeded per well in 12-well plates and were grown for 1 day in phenol red-free medium, containing 5% DCC-FC. Cells were then treated with 20 μg/ml C3 exoenzyme (produced from the pGEX2T-C3 plasmid; kindly provided by Dr LA Feig, Tufts University School of Medicine, Boston, MA, USA) in a final volume of 0.5 ml [21]. Cells received E2 (5 × 10^-9 ^M) 24 hours later and lysates were obtained after a further 16 hours for the luciferase assay as already described.

### Statistical analysis

Statistical analysis of the data was conducted using an unpaired two-sample *t *test. Significance was defined as *P *< 0.02 or *P *< 0.05, as indicated in the text.

## Results

### Prenyltransferase inhibitors stimulate ERE-dependent luciferase activity in stably transfected MCF-7 cells but not in transiently transfected MCF-7 cells

**Figure 1:**
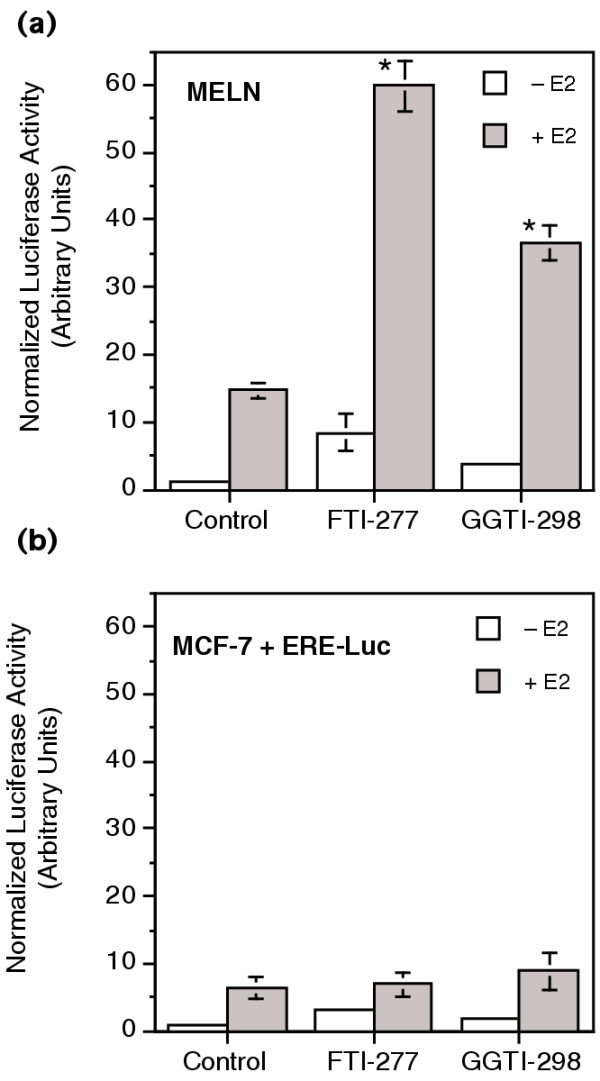
Effects of prenyltransferase inhibitors on estrogen response element (ERE)-dependent luciferase activity **(a) **in stably transfected MELN cells and **(b) **in transiently transfected MCF-7 cells. Both MELN cells and MCF-7 cells are deprived of estradiol (E2) for 4 days, and the MCF-7 cells are transiently transfected with the ERE-β-glob-Luciferase and the Renilla luciferase plasmids. Three hours after transfection, cells were treated or not with FTI-277 (10 μM or dithiothreitol/dimethylsulfoxide vehicle). Twenty-four hours later, cells were stimulated with E2 (5 nM) or ethanol and were treated or not with FTI-277 or GGTI-298 (10 μM or 5 μM, respectively, or dithiothreitol/dimethylsulfoxide vehicle). Luciferase activity was quantified 16 hours after E2 addition, as described in Materials and methods. Results are expressed in arbitrary units after normalization. Error bars indicate the mean values ± standard deviation from triplicate experiments, and the results are representative of two independent experiments. Results obtained show that prenylation inhibitors statistically increase the luciferase activity induced by E2 compared with the activity induced by E2 alone in MELN cells only (* *P *< 0.02).

We previously showed that both FTI-277 and GGTI-298 markedly enhance ER-mediated transcription in MELN cells [17], a clone of MCF-7 cells stably transfected with the ERE-β globin-luciferase reporter gene (Fig. 1a). After 5 days of E2 deprivation, cells were treated or not with 5 nM E2, in the presence of or in the absence of prenyltransferase inhibitors, and the luciferase activity was quantified 16 hours later. For optimal FTI-277 treatments, a 24-hour preincubation was systematically performed before E2 addition, whereas GGTI-298 was added simultaneously with E2 [17]. We confirmed the efficiency and specificity of FTI-277 and GGTI-298 to inhibit protein prenylation in MELN cells in these experimental conditions. Indeed, for this purpose, we checked whether HDJ2, a farnesylated protein, and Rap1A, a geranylgeranylated protein, were indeed not prenylated in the presence of 10 μM FTI-277 and 5 μM GGTI-298, respectively (data not shown).

E2 alone induced a 14.7-fold increase in the luciferase activity in MELN cells. In the absence of E2 (Fig. 1a, white bars), FTI-277 and GGTI-298 stimulated the basal transcriptional activity by 8.4-fold and 3.9-fold, respectively. In the presence of E2 (Fig. 1a, grey bars), FTI-277 and GGTI-298 dramatically enhanced the ability of E2 to stimulate transcription by an additional 4.1-fold for FTI-277 and 2.5-fold for GGTI-298. The ability of E2 to stimulate transcription was statistically enhanced by both FTI-277 and GGTI-298 (*P *< 0.02).

In parallel to this cell model, in which the Luciferase gene is stably integrated into the genome, we determined the effects of prenylation inhibitors in MCF-7 cells that were transiently transfected with the ERE-β-glob-Luciferase plasmid (Fig. 1b). In vehicle-treated cells, E2 still induced a 6.4-fold induction of luciferase activity. In the absence of E2, FTI-277 and GGTI-298 only stimulated the basal transcriptional activity by 3.2-fold and twofold, respectively. In the presence of E2, the transcription was only enhanced by an additional 1.1-fold for FTI-277 and 1.4-fold for GGTI-298. The ability of E2 to stimulate transcription was not statistically enhanced by FTI-277 or by GGTI-298 (*P *< 0.05). This result outlines the necessity of having a stably integrated reporter gene in order to elicit the effects of prenylation inhibitors.

**Figure 2:**
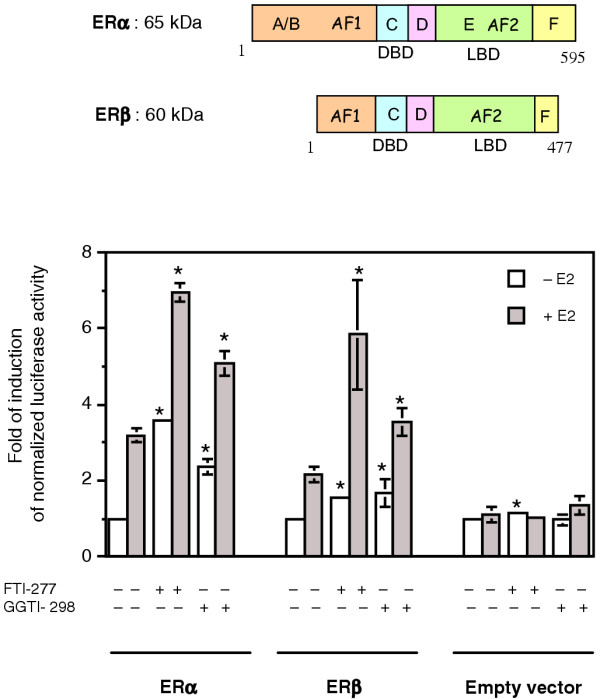
Effects of prenyltransferase inhibitors on estrogen response element-dependent luciferase activity in HELN cells transfected with estrogen receptor (ER) α or ERβ. Cells, deprived of estradiol (E2) for 4 days, were co-transfected with the Renilla luciferase plasmid and either HEG0 (full-length ERα), pSG5-REβ (full-length ERβ) or pSG5 (empty vector). Five hours after transfection, cells were treated or not with FTI-277 (10 μM or dithiothreitol/dimethylsulfoxide vehicle), and 24 hours later they were stimulated with E2 (5 nM) or ethanol and were treated or not with FTI-277 or GGTI-298 (10 μM or 5 μM, respectively, or dithiothreitol/dimethylsulfoxide vehicle). Luciferase activity was quantified 16 hours after E2 addition, as described in Materials and methods. Results are expressed in arbitrary units after normalization. Error bars indicate the mean values ± standard deviation from triplicate experiments, and the results are representative of three independent experiments. Results obtained show that prenylation inhibitors statistically increase the luciferase activity on their own compared with control cells (white bars) and statistically increase the luciferase activity induced by E2 compared with the activity induced by E2 alone (grey bars) in MELN cells transfected with ERα or ERβ (* *P *< 0.05).

This necessity was confirmed by checking the effects of FTI-277 in two other cell lines having a stably integrated reporter system, beside the MELN cells. We used the MVLN cells (MCF-7 cells stably transfected with an ERE-vit-tk-luc plasmid [22]; data not shown) and HELN cells (HeLa cells stably transfected with the ERE-β-globin-luc-SV-Neo plasmid and transiently transfected with the ERα, as described in Fig. 2). The data clearly indicate that in the three models the farnesyltransferase inhibitor does stimulate the ERE-dependent luciferase activity, in the absence of and in the presence of E2.

### Prenyltransferase inhibitors stimulate both ERα-mediated or ERβ-mediated ERE-dependent luciferase activity in HELN cells

We then examined whether the effects of prenylation inhibitors were preserved in cells that stably expressed the gene coding the ERE-β glob-Luciferase but only transiently expressed the ER. For this purpose we used HELN cells, which are derived from the HeLa human cervical cancer cell line. HELN cells have no endogenous ER but stably express the gene coding the ERE-β glob-Luciferase [19]. As expected, we observed no induction of luciferase activity by E2 in cells transfected with the empty vector (Fig. 2). For all experiments, luciferase activity was normalized according to the expression of the co-transfected Renilla Luciferase gene so as to take into account the variations due to transfection efficiency.

HELN cells were then transfected with either ERα or ERβ. In both cases, E2 induced the luciferase activity in vehicle-treated cells (3.2-fold and 2.2-fold, for ERα-transfected and ERβ-transfected cells, respectively), which was lower than the activity obtained in MELN cells or MCF-7 cells (14.7-fold and 6.4-fold, respectively). In the absence of E2 (Fig. 2, white bars), FTI-277 and GGTI-298 only weakly stimulated the basal transcriptional activity by 3.6-fold and 2.4-fold, respectively, for ERα and by 1.5-fold and 1.7-fold, respectively, for ERβ. These stimulations were statistically significant (*P *< 0.05). In the presence of E2 (Fig. 2, grey bars), FTI-277 and GGTI-298 enhanced the basal transcriptional level by an additional 2.2-fold and 1.6-fold, respectively, for ERα and by an additional 2.7-fold and 1.6-fold, respectively, for ERβ. Altogether, the effect of E2 was significantly enhanced by both prenylation inhibitors with either ERα or ERβ (*P *< 0.05). As previously described for MELN cells [17], these effects were strongly inhibited by ICI 182,780 (data not shown).

### Role of AF-1 and AF-2 in the effect of prenyltransferase inhibitors on ERE-dependent luciferase activity in HELN cells

In order to understand how prenylated proteins regulate ER transcription, we investigated whether the effects of prenylation inhibitors on transcription involve the AF-1 and/or AF-2 functions of the ERα. For this purpose, we used HELN cells that stably expressed the ERE-β glob-Luciferase reporter gene and were transiently transfected with plasmids coding either the full-length human ERα (HEG0) or the ERα mutants with defective AF-1 (HEG19) or AF-2 (HEG7) activities [20].

**Figure 3:**
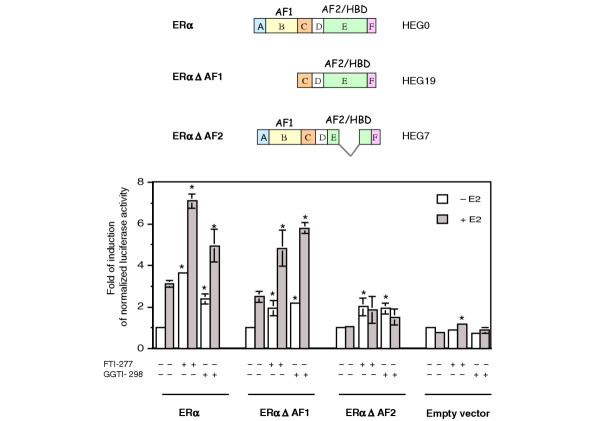
Effects of prenyltransferase inhibitors on estrogen response element-dependent luciferase activity in HELN cells, transfected with estrogen receptor (ER) α or its mutants. Cells, deprived of estradiol (E2) for 4 days, were co-transfected with the Renilla luciferase plasmid and either HEG0 (full-length ERα), HEG19 (AF-1-deleted ERα), HEG7 (AF-2-deleted ERα) or pSG5 (empty vector). Five hours after transfection, cells were treated or not with FTI-277 (10 μM or dithiothreitol/dimethylsulfoxide vehicle), and 24 hours later they were stimulated with E2 (5 nM) or ethanol and treated or not with FTI-277 or GGTI-298 (10 μM or 5 μM, respectively, or dithiothreitol/dimethylsulfoxide vehicle). Luciferase activity was quantified 16 hours after E2 addition, as described in Materials and methods. Results are expressed in arbitrary units after normalization. Error bars indicate the mean values ± standard deviation from triplicate experiments, and the results are representative of three independent experiments. Results obtained show that prenylation inhibitors statistically increase or do not statistically increase the luciferase activity on their own compared with control cells (white bars) and statistically increase the luciferase activity induced by E2 compared with the activity induced by E2 alone (grey bars) in HELN cells transfected with ERα or its mutants (* *P *< 0.02).

Cells, deprived of E2 for 4 days, were co-transfected with the Renilla luciferase plasmid and the plasmid coding full-length ERα or its mutants (Fig. 3). As expected, E2 alone could induce the luciferase activity only in cells transfected with plasmids coding either the full-length human ERα (HEG0) or the AF-1-defective mutant (HEG19). Indeed, AF-1 is constitutively active, is ligand independent and is mainly induced by growth factors. The absence of induction in cells transfected with the plasmid coding the AF-2 defective mutant (HEG7) is in agreement with the ligand dependence of AF-2.

In the presence of E2 (Fig. 3, grey bars), FTI-277 and GGTI-298 induced a statistically significant increase in the stimulation of transcription by E2 by 2.3-fold and 1.6-fold, respectively, in cells transfected with the full-length ERα (HEG0) and by 1.9-fold and 2.3-fold, respectively, in cells transfected with the AF-1 defective mutant (HEG19) (*P *< 0.02). In the absence of E2 (Fig. 3, white bars), FTI-277 and GTI-298 statistically increased the transcription of the luciferase gene in cells transfected with plasmids coding either the full-length human ERα or the two defective mutants by twofold to threefold. Cells transfected with the plasmid coding the AF-2 defective mutant (HEG7) exhibited no significant induction of luciferase activity in the presence of the prenylation inhibitors compared with E2 alone. The induction observed in cells transfected with the empty vector in the presence of FTI-277 had no biological significance as the final induction level was very low (1.2-fold).

### Decreased cytoplasmic and nuclear presence of ERα due to prenyltransferase inhibitors in MCF-7 cells

**Figure 4:**
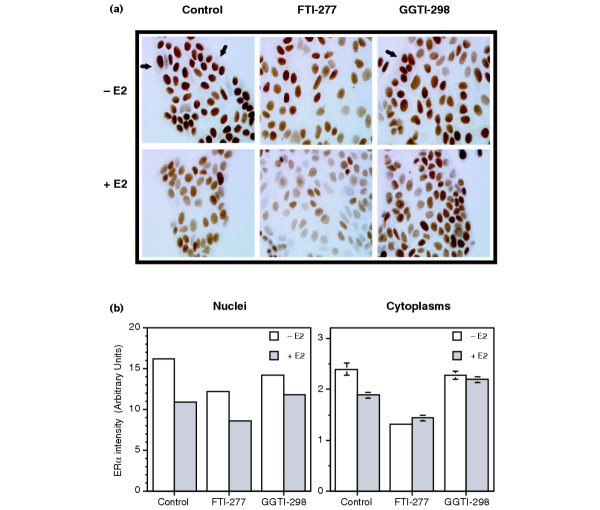
Immunocytochemical analysis of the effects of prenyltransferase inhibitors on estrogen receptor (ER) α distribution in MCF-7 cells. **(a) **Cells, deprived of estradiol (E2) for 7 days, were treated or not with FTI-277 (10 μM or dithiothreitol/dimethylsulfoxide vehicle), and 24 hours later they were stimulated with E2 (5 nM) or ethanol and treated or not with FTI-277 or GGTI-298 (10 μM or 5 μM, respectively, or vehicle). After 24 hours, cells were fixed and stained. One randomly selected field is presented for each treatment. **(b) **Data were quantified by determining the grey value of both the nucleus and the cytoplasm for each cell counted, as described in Materials and methods. For each experimental condition, six randomly selected fields were analyzed. The total number of cells present in the fields ranged from 250 to 400. Error bars indicate the mean values ± standard error of the mean, and the results are representative of two independent experiments.

To investigate the effects of prenylation inhibitors on ERα localization within the cell, we detected the presence of ERα by immunocytochemistry in MCF-7 cells, treated or not with E2 and prenylation inhibitors for 24 hours. The result of this experiment is shown in Fig. 4a, with the relative quantification presented in Fig. 4b.

As expected, ERα is highly concentrated in the nuclei of the untreated control with a significant staining of the corresponding cytoplasms (indicated by arrows in Fig. 4a). In the presence of E2, the staining intensity of both nuclei and cytoplasms was clearly decreased, with no distinct staining of the cytoplasms. In the absence of E2, similar decreases in labeling intensity were observed in the cytoplasms and nuclei of FTI-277-treated cells. Indeed, the presence of FTI-277 dramatically enhanced the ability of E2 to decrease nuclear staining, with the persistent absence of cytoplasmic staining. GGTI-298 similarly induced a decrease of ERα staining intensity in the cell nuclei, although to a lesser extent than did FTI-277, both in the presence of and in the absence of E2. Unexpectedly, no significant decrease in the staining intensity was observed in the cytoplasms of GGTI-298 treated cells, either in the presence of or in the absence of E2.

It must be outlined that these effects were observed after 24 hours of treatment with the prenylation inhibitors, whereas no effect was observed after short-term incubation (2 hours; data not shown).

### Involvement of Rho proteins in ERE-dependent luciferase activity in MELN cells

**Figure 5:**
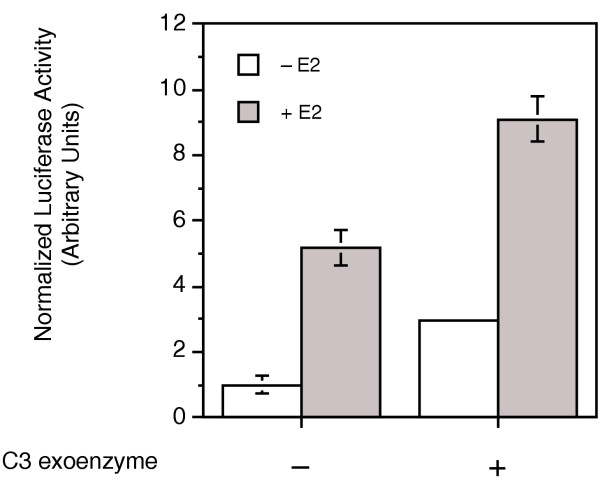
Effect of C3 exoenzyme on estrogen response element-dependent luciferase activity in MCF-7 cells. Cells, deprived of estradiol (E2) for 4 days, were treated with C3 exoenzyme as described in Materials and methods. Twenty-four hours later the cells were stimulated with E2 (5 nM) or ethanol. Luciferase activities were quantified 16 hours after E2 addition, as described in Material and methods. Results are expressed in arbitrary units after protein normalization. Error bars indicate the mean values ± standard deviation from triplicate experiments, and the results are representative of two independent experiments.

*C. botulinum *C3 exoenzyme, a specific inhibitor of RhoA, RhoB and RhoC proteins, was used to determine whether the effects of prenylation inhibitors on ERE-mediated transcription were due to Rho proteins. After 4 days of E2 deprivation, cells were treated or not with C3 exoenzyme. E2 was added after 24 hours, and the luciferase activity was quantified 16 hours later (Fig. 5). The inhibition of ADP-ribosylation had already been examined under these conditions, demonstrating that the C3 exoenzyme does enter the treated cells (data not shown). The results clearly show an induction of luciferase activity by the C3 exoenzyme, in the presence of or in the absence of E2. This demonstrates the involvement of Rho proteins in the negative control of the ERE-dependent luciferase activity.

## Discussion

Activation of ERs by estrogens triggers both ER nuclear transcriptional activity and the Src/Ras/Erks pathway-dependent mitogenic activity [9,13]. Prenylated proteins have been implicated in both estrogenic actions [17,23], and the prenylated Rho GTPases are now considered important modulators of ER transcriptional activity [24,25]. We previously demonstrated that prenylated proteins antagonize the ability of ERα to stimulate ERE-dependent transcriptional activity, potentially through the recruitment of a co-regulator [17]. In the present report, we proved that the stable integration of a plasmid coding the ERE-dependent luciferase in MCF-7 cells, or in HeLa cells transfected with the ERα gene, is necessary for FTI-277 and GGTI-298 effects. We did, however, observe a significant but expected stimulation of luciferase activity by E2 alone in the transiently transfected cells.

The need for a stable integration of the reporter system suggests that integration in the cell genome, an optimal chromatin environment of the ERE, and a precise low number of copies of the gene of interest are relevant conditions for the effects of the prenylation inhibitor, and consequently for the prenylated protein role. More insight in the interactions between the co-regulator complex in chromatin and prenylated proteins will be possible by performing chromatin immunoprecipitation experiments.

We chose HELN cells as a model of ER-free cells. These cells are stably transfected with the plasmid coding the ERE-dependent luciferase. Neither E2 nor prenylation inhibitors had any effect on the luciferase activity in these cells. In contrast, the ERE-dependent activation of transcription induced in the presence of both prenyltransferase inhibitors and reversed by the antiestrogen ICI 182,780 (data not shown) could be observed after transient transfection of HELN cells with the ERα gene. The classical consensus ERE is directly bound to ERα and ERβ, and at this site ERβ is a weaker transactivator [3-5]. In the presence of E2 and either ERα or ERβ, the comparison of the effects of prenylation inhibitors revealed that prenylated proteins had similar effects on the transcriptional activities of both ERs. These results are in agreement with the fact that Rho GDIα, a Rho guanine dissociation inhibitor that represses the activity of Rho GTPases including RhoA, Rac1 and Cdc42, has been shown to be a positive regulator of both ERα and ERβ transcriptional activities in human osteosarcoma cells [24]

It must be outlined, however, that there are important DNA binding sites for ERs other than ERE [26,27] where ERβ exerts different, or sometimes even opposite, effects to ERα (e.g. at AP-1 and Sp-1 sites) [28,29]. Such differential signaling between ERα and ERβ may exist with estrogen and prenylated proteins at the AP-1 response element. As it has been shown that AP1-mediated transcription is downregulated by RhoB, then RhoB may to some extent act on E2-mediated effects through this pathway [30]. Moreover, the activity of a number of transcription factors that may determine the overall promoter activity of ERE-containing genes is known to be altered by prenylated proteins (e.g. SRF, NF-κB) [31-34].

Our data strongly suggest the involvement of AF-2 in the transcriptional activation induced by prenylation inhibitors. Indeed, significant luciferase activity in cells expressing the AF-1 deleted mutant is observed in the presence of inhibitors and in both the presence of and the absence of E2, whereas the rates of induction observed in the AF-2-deleted mutant in the presence of inhibitors are similar in the presence of and the absence of E2. This is confirmed by the fact that the effects of prenylation inhibitors are similar on ERα and ERβ, whereas ERβ seems to have no significant AF-1 activity and thus depends entirely on the ligand-dependent AF-2 [2]. Interestingly, prenylated proteins appear to also regulate ER in an AF-2-independent manner, as the AF-2-deleted mutant did not completely abolish the increase in ER transcriptional activity induced by either FTI-277 or GGTI-298, with a significant stimulation in the absence of E2. This is in agreement with the data suggesting that induction of ER transactivation by RhoGDI is mediated largely by an ER AF-2-dependent mechanism, and to a lesser extent by an AF-2-independent mechanism [25].

The process by which a portion of the classical ER relocates to the cell membrane remains undetermined. Steroid receptors rapidly shuttle between the nucleus and cytoplasm by active nuclear import and export mechanisms. In the absence of identified post-translational modifications that could be involved in the attachment of the ER to the cell membrane, it is probable that membrane translocation of the ER is facilitated by another protein such as caveolin-1, which has been reported to interact with the ER [35]. Indeed, proteins of the Rho family (RhoA and Rac1) are targeted to caveolae in fibroblasts and are retained there by an unknown mechanism [36]. Moreover, p122, a GTPase activating protein is localized in the caveolae of both fibroblastic and epithelial cells, and plays an important role in caveolin distribution through the reorganization of the actin cytoskeleton [37].

The intracellular localization of MNAR and the way it traffics around the cell with the ER is still to be determined. Prenylated proteins may then modulate ER signaling through each key step in the various pathways: membrane attachment, translocation to the nucleus, stability, and genomic or nongenomic activities. The mechanisms that are responsible for the effects of prenylation inhibitors could involve modifications in the intracellular localization, or the level of ER protein expression, which would facilitate transcription. As an example, RhoA is a geranylgeranylated protein known to stimulate the degradation of the cyclin-dependent kinase inhibitor, P27^kip^. We previously demonstrated that the ER protein and RNA expression levels in total cell lysates are decreased by both FTI-277 and GGTI-298 treatment [17], and that ICI 182,780, a pure estrogen antagonist, induces the rapid relocation of ERα to an insoluble nuclear fraction, followed by its degradation [38]. Furthermore, the association of the SRC-1 co-activator to the ER is increased by FTI-277 and GGTI-298 [17], and slows the cytoplasm to nucleus mobility of ligand–ER complexes [39].

Immunocytochemical analysis showed a clear decrease in the intensity of ER staining in the nuclei of MCF-7 cells treated with either E2 or with FTI-277, or both, with a concomitant decrease in cytoplasmic ER staining. It is important to note that GGTI-298 has a much weaker effect, mainly in the cytoplasm. This suggests that farnesylated proteins predominantly maintain a high level of ER expression within the whole cell (either by repressing the degradation of ER or by increasing its expression or stability), whereas geranylgeranylated proteins could only stimulate the nuclear expression of the ER. Such a mechanism must be elucidated; indeed, the degradation of ERα induced by E2 is dependent on the proteasome [40], as has already been suggested for RhoB [41]. Since prenylation inhibitors had no effect on ER localization after a short incubation period in MCF-7 cells (2 hours; data not shown), we speculate that prenylated inhibitors act mainly by inhibiting active regulatory prenylated protein rather than by activating inhibited proteins.

Our data strongly suggest that both farnesylated proteins and geranylgeranylated proteins modulate ER-mediated activities, through either a common pathway or a distinct pathway. A special emphasis has already been placed on the role of Rho GTPases, and especially RhoA, Rac1 and cdc42, as ER-negative modulators in human osteosarcoma and breast cancer cells [24]. RhoA may therefore be the target of GGTI-298, explaining part of the described effects. We evaluated the role of the RhoA, RhoB, or RhoC proteins in the negative regulation of ER transcriptional activity, using the C3 exoenzyme. This toxin inhibits the three small GTPases through ADP-ribosylation. We could therefore implicate Rho proteins, which are generally geranylgeranylated, as negative modulators of ER transcriptional activity in MCF-7 cells. As both farnesylated proteins and geranylgeranylated proteins play a role in the negative control of ER-mediated transcriptional activity, many farnesylated proteins are potential candidates for the stimulatory effects of FTI-277. Due to the absence of specific inhibitors of the GTPase activity of farnesylated proteins, a more progressive screening of the numerous farnesylated proteins is required to identify those that are involved in the negative regulation of ER-mediated transcriptional activity.

It is important to note that RhoB can be both farnesylated and geranylgeranylated, which modifies the cellular localization of the protein. Indeed, farnesylated RhoB is located at the plasma membrane and geranylgeranylated RhoB is endosomal [42]. An increase in RhoB mRNA is observed in some breast cancer cell lines [43]. In rat fibroblasts, it has been shown that in the presence of farnesyltransferase inhibitors, the antiproliferative effect is driven by the geranylgeranylated form of RhoB [44]. Rho proteins and other prenylated proteins may then modulate ER-mediated transcription. Apart from the involvement of RhoGDI as a positive regulator of ER transactivation, it has been shown that Brx, a guanine nucleotide exchange factor for Rho proteins and member of the Dbl oncogene family, contains a nuclear hormone receptor binding region. Brx affects ER-mediated gene activation by a mechanism that is dependent on the Cdc42Hs signaling pathway [45]. Moreover, constitutively active forms of c-Raf and Rac synergistically enhance the CREB-binding protein/p300-mediated increase of transcription in T-cell activation signals [46]. Finally, a constitutively active form of Cdc-42 induces H4 hyperacetylation in chromatin [47].

Altogether, our data strongly suggest that several prenylated proteins, either farnesylated or geranylgeranylated, modulate ER signaling by repressing its transcriptional activity and/or increasing its stability. The determination of the exact Rho protein(s) involved in the effect we described is a major step of the research we are currently performing in the laboratory, in addition to the identification of the potential ER co-regulators.

## Conclusion

Targeting the estrogen signaling pathway has proved to be of great value in the treatment of human breast cancer, and current evidence suggests that such targets hold great potential in the prevention of human breast cancer. Evaluation of the multiple pathways that can cross-talk with estrogen signaling pathways should help improve our understanding of some of the possible mechanisms of *de novo *and acquired tamoxifen resistance. Farnesyltransferase inhibitors are a new class of anticancer drugs that are at present in phase III clinical evaluation [48-50]. We previously showed that the combination of a farnesyltransferase inhibitor and tamoxifen may increase the antitumor effect of either drug alone in breast cancer [23]. Gaining further insight into the involvement of prenylated proteins in the estrogen signaling pathways should allow a more rational approach to treating and/or preventing hormone-resistant phenotypes.

## Abbreviations

DCC-FCS = dextran-coated charcoal treated fetal calf serum; DMEM = Dulbecco's modified Eagle's medium; E2 = estradiol; ER = estrogen receptor; ERE = estrogen response element; FCS = fetal calf serum; FTI-277 = farnesyltransferase inhibitor; GGTI-298 = geranylgeranyltransferase inhibitor; MNAR = modulator of non-genomic activity of estrogen receptor; PBS = phosphate-buffered saline.

## Competing interests

The author(s) declare that they have no competing interests.

## Authors' contributions

The authors' contributions to this research are reflected in the order shown, with the exception of SDS who supervised the research and the preparation of this report. PC, GS, CMG and SDS carried out the experiments and PC drafted the manuscript. PB provided the MELN cells and the HELN cells, and provided technical support to initiate the study. PR carried out the immunocytochemical labeling. SDS and JCF conceived of the study, and participated in its design and coordination. GF, head of the department, gave helpful advice during the whole process. SDS performed the statistical analysis. All authors read and approved the final manuscript.
